# Substance Use Disorder Diagnoses Among Older Medicare Beneficiaries Hospitalized With Traumatic Brain Injury

**DOI:** 10.1093/geroni/igaf122.3660

**Published:** 2025-12-31

**Authors:** Yoon Chung Kim

**Affiliations:** University of Maryland School of Medicine, Baltimore, Maryland, United States

## Abstract

Traumatic brain injury (TBI) is a common, fall-related injury among older adults. Studies have reported high rates of pre-existing alcohol and/or substance use disorders (AUD/SUD) among younger adults with TBI, however, less is known about AUD/SUD among older adults with TBI. We estimated the prevalence of pre-existing AUD/SUD among older adults hospitalized with TBI and identified demographic and clinical characteristics associated with AUD/SUD status. We conducted a retrospective cohort study using Medicare administrative claims data, including beneficiaries aged ≥65 years hospitalized with TBI between 2010-2017. Pre-existing AUD/SUD was defined using diagnostic codes during the 6 months pre-TBI and 3 months post-TBI. Among beneficiaries hospitalized with TBI (n = 20,350), prevalence of pre-existing AUD/SUD diagnoses was 8.4% (n = 1,713). AUD diagnoses were most common (7.1%); 1.8% had SUD diagnoses. Among those with any AUD/SUD, 84.8% had AUD and 20.9% had ≥1 SUD diagnoses. Opioid use disorder was the most common SUD (10.4% SUD diagnoses). Compared to Medicare beneficiaries without an AUD/SUD diagnosis, beneficiaries with a pre-existing AUD/SUD diagnosis had lower odds of being older (odds ratio (OR) 0.91; 95% confidence interval (CI): 0.90, 0.92), being female (OR 0.53; 95% CI 0.48, 0.59), higher odds of having chronic obstructive pulmonary disease (OR 2.04; 95% CI 1.82, 2.28) or depression (OR 1.50; 95% CI 1.34, 1.67), and higher odds of dual eligibility for Medicare and Medicaid (OR 1.26; 95% CI 1.12, 1.42). Older Medicaid beneficiaries hospitalized with TBI who have pre-existing AUD/SUD have unique clinical complexities and may benefit from integrated and targeted treatments.

